# BUILDING A CONTINUOUS DEMENTIA MANAGEMENT MODEL IN COMMUNITIES OF SHANGHAI

**DOI:** 10.1093/geroni/igz038.1665

**Published:** 2019-11-08

**Authors:** Xia Li, Qi Qiu, Yinghua Yang, Ling Sun, MinJun Jiang, chunling Gu, Ming Cui, Xiang Lin

**Affiliations:** 1 Shanghai Mental Health Center, Shanghai, Shanghai, China; 2 Shanghai Mental Health Center, Shanghai, Shanghai, China (People’s Republic)

## Abstract

Over 10 million people with Alzheimer’s disease or related dementias (ADRD) live in China. In Shanghai, the prevalence of ADRD is about 3-4% among aged 60 or older, and approximately 70-85% have never been diagnosed. This study reported the pilot testing results of a dementia management model launched by Shanghai Mental Health Center to build dementia friendly communities. The dementia management model links screening, diagnosis, care planning, treatment, and services, to improve dementia literacy and standard diagnosis rate. About 3,786 senior residents were screened using the AD 8 and MoCA scales. The cognitively intact group was suggested for annual check-up, while at -risk groups were referred to formal diagnosis and intervention. About 125 older adults with a mild cognitive impairment diagnosis were provided referrals for cognitive training, and 109 diagnosed with dementia were provided medical and social interventions. This management model adds to dementia awareness and education.

